# Association of Urinary Mycotoxins with Sperm Quality: A Case-Control Study in Southern Nigeria

**DOI:** 10.3390/toxins16030119

**Published:** 2024-02-29

**Authors:** Friday Ebhodaghe Okonofua, Lorretta Favour Chizomam Ntoimo, Emmanuel Iyayi Unuabonah, Titus Afred Makudali Msagati, Oladiran Ayodeji, Michael Aziken, Akhere Omonkhua, Victor Ohenhen, Celestina Olafusi, Moses O. Alfred

**Affiliations:** 1 Centre of Excellence in Reproductive Health Innovation (CERHI), University of Benin, Benin City 300213, Nigeria; lorretta.ntoimo@fuoye.edu.ng (L.F.C.N.); akuekegbe.omonkhua@uniben.edu (A.O.); 2 Department of Obstetrics and Gynaecology, University of Benin Teaching Hospital, University of Benin, Benin City 300213, Nigeria; michael.aziken@uniben.edu; 3 Women’s Health and Action Research Centre (WHARC), Benin City 300283, Nigeria; 4 Department of Demography and Social Statistics, Federal University Oye-Ekiti, Oye-Ekiti 371104, Nigeria; 5 Department of Chemical Sciences, Redeemer University, Ede 232101, Nigeria; unuabonahe@run.edu.ng (E.I.U.); alfredm@run.edu.ng (M.O.A.); 6 African Centre of Excellence for Water and Environmental Research (ACEWATER), Redeemer’s University, Ede 232101, Nigeria; 7 Institute for Nanotechnology and Water Sustainability, College of Science, Engineering & Technology, University of South Africa, The Science Campus, Corner Christiaan De Wet and Pioneer Drive, Roodepoort, Johannesburg 1709, South Africa; msagatam@unisa.ac.za; 8 Department of Obstetrics and Gynaecology, University of Medical Sciences, Ilaje, Ondo 351104, Nigeria; ladiran54@gmail.com; 9 Department of Medical Biochemistry, School of Basic Medical Sciences, University of Benin, Benin City 300213, Nigeria; 10 Department of Obstetrics and Gynaecology, Central Hospital, Benin City 300102, Nigeria; victorohenhen@gmail.com; 11 Department of Biochemistry, Faculty of Basic Medical Sciences, University of Medical Sciences, Ilaje, Ondo 351104, Nigeria; olafusicelestina@gmail.com

**Keywords:** male infertility, urinary mycotoxins, Nigeria, case-control study, sperm count

## Abstract

The objective of this study was to determine the association between mycotoxins and the quality of spermatozoa in Nigeria. We designed a prospective case-control study involving 136 men diagnosed with reduced sperm count and quality in five infertility clinics in southwest Nigeria and 154 normal fertile controls. Sperm analysis was conducted in accordance with the recommendations of the World Health OrganizationWHO, while Liquid Chromatography–Mass Spectrometry was used to assay three metabolites of mycotoxins (zearalenone, ochratoxin A, and deoxyvinelol) in the urine samples of cases and controls. The data were analysed with descriptive statistics and non-parametric linear regression. The results showed no overall significant difference in levels of these metabolites between the cases and control groups. In contrast, higher levels of zearalenone and ochratoxin A significantly decreased sperm motility in the cases. Similarly, an increase in the level of ochratoxin A decreased sperm morphology in the unadjusted model in the cases. We conclude that exposure to mycotoxins reduces the quality of spermatozoa (motility and morphology) in Nigerian men but may have no effect on sperm count. Efforts to reduce the exposure of men to mycotoxins are important interventions to improve sperm quality and reduce the prevalence of male infertility in the country.

## 1. Introduction

Available evidence indicates that there is an ongoing decline in total sperm count and good-quality spermatozoa in many countries around the world [1], including the African region [2]. This decreasing count and quality of sperm has resulted in the World Health Organization revising its criteria for normal sperm count [3], and it is likely the cause of the increasing rate of male infertility globally and in the African region [4]. Several publications present evidence of an increasing rate of male infertility in many parts of Africa [5,6,7] most often attributed to low sperm count and poor-quality spermatozoa. In Nigeria, the prevalence of male-factor infertility as compared to female-factor infertility in published studies ranges from 40% to 60% [8,9,10,11], with an increasing proportion of cases where the male factor is reported as the dominant cause of infertility. 

To date, the causes of male infertility and the increasing rate of male infertility in the African region have not been well elucidated. Previous studies have identified plausible risk factors to include epidemiological factors [11,12], behavioural factors (smoking, alcohol, etc.) [11,13], genetic factors [14,15], and exposures to environmental toxins [16,17,18]. Our recent scoping review of published studies [19] summarized the leading risk factors associated with male infertility in Africa as “biological/physiological/genetic, behavioural/lifestyle, environmental factors, and sociodemographic risk factors”. To date, none of these hypothesised risk factors and causes have been confirmed or substantiated. Environmental risk factors are particularly worrying, given the high rate of environmental contamination in many African countries. Several environmental factors that have been hypothesized to be associated with male infertility in Africa include exposure to heavy metals and exposure to food contaminated with aflatoxins (mycotoxins) [20,21,22,23]. In a previous commentary, we raised the possibility that male infertility may be associated with the intake of food contaminated with mycotoxins [24]. Available evidence indicates that various metabolites of mycotoxins may lower sperm count in animals [21,25] but the evidence has not been convincingly demonstrated in humans. Mycotoxins frequently contaminate a large proportion of food and food products due to poor storage facilities [26,27], and these foods are frequently consumed in Nigeria without recognition of the possible harmful effects of the mycotoxins contained in them. 

The objective of this study was to determine the association between exposure to mycotoxins, sperm quality, and male infertility in Nigeria. Our research was designed to test the null hypothesis that men diagnosed with poor-quality sperm in infertility clinics in southern Nigeria do not differ in urinary levels of metabolites of mycotoxins as compared to those with normal sperm count and good-quality sperm attending the same clinics. We believe that the results of this study will enable the identification of relevant policies and programs to prevent male infertility in Nigeria, with possible implications for other African countries.

## 2. Results

**Figure 1:**
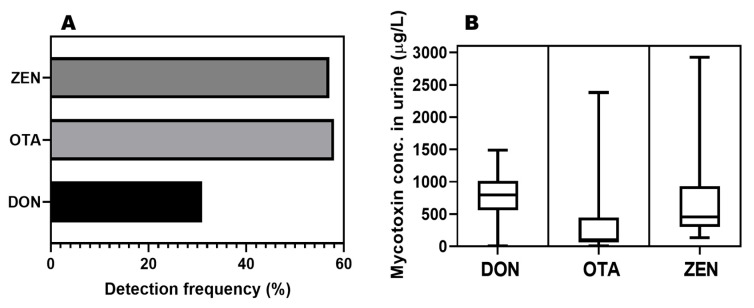
(**A**) Plot of detection frequency and (**B**) box–whisker plots of mycotoxin data from urine analysis (DON = deoxynivalenol; OTA = ochratoxin A; ZEN = zearalenone).

**Figure 2:**
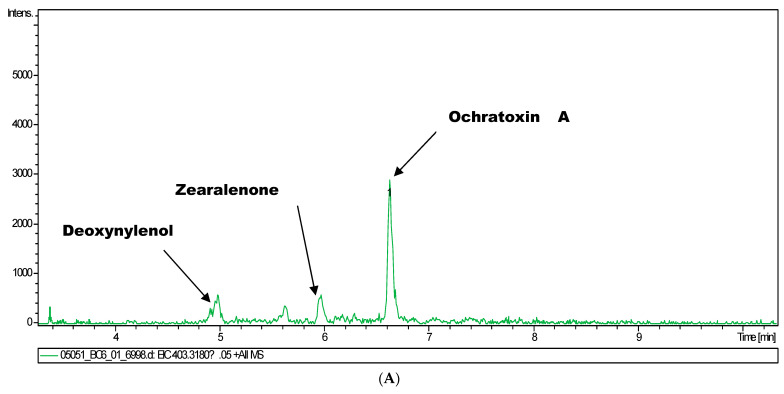

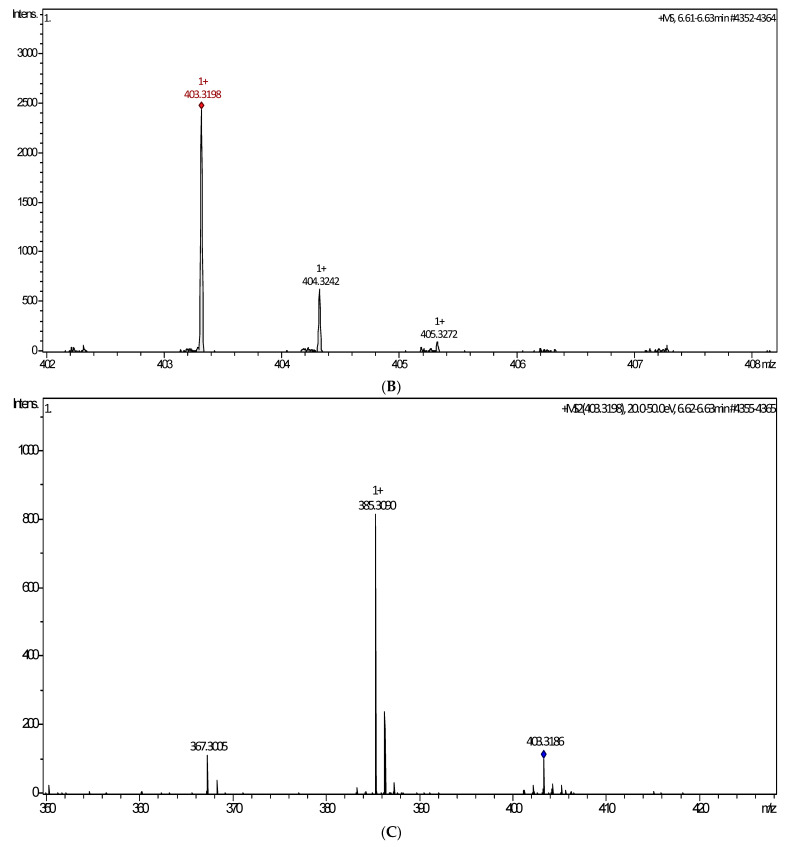
(**A**) LC-MS-MS Chromatograms for urine sample. (**B**) MS Spectra of Ochratoxin A for urine sample 05051. (**C**) MS^2^ spectra for Ochratoxin A for urine sample.

Considering the data for all the urine samples collected for analysis, it is observed that ochratoxin A and zearalenone are more frequently detected in urine samples than deoxynivalenol (31%) (Figure 1A). The box–whisker plots reveal that more data for OTA and ZEN are found in the 25th percentile region with ZEN showing the highest toxin concentration in urine samples (Figure 1B). The geometric mean of these mycotoxins in urine samples collected for this study follow the order: deoxynivalenol (544.4 ± 3.34 µg/L) > zearalenone (466.8 ± 1.85 µg/L) > ochratoxin A (149.6 ± 3.90 µg/L) as shown in Figure 2. 

The distributions of the case and control groups by their socio-demographic and behavioural characteristics are presented in Table 1. The study population was not statistically different between the case and control groups in all the socio-demographic and behavioural characteristics except in their BMI. There is no statistically significant difference in socio-demographic and behavioural characteristics between the respondents whose urine underwent biochemical analysis (n = 179) and those whose urine did not (n = 111), except in religion. A sub-group descriptive analysis for those 179 participants shows no statistically significant difference in socio-demographic and behavioural characteristics between the case and control groups.

The distribution of the cases and control by sperm parameters is presented in Table 2. The two groups varied significantly in sperm count and quality. Variations in the metabolites of mycotoxins are presented in Table 3 by fertility status (case and control groups). The distribution of the metabolites of mycotoxins was not statistically different between the case and control groups. However, the median value of zearalenone was higher in the cases compared to the control group. 

### 2.1. Variation in the Metabolites by Sperm Parameters

The level of zearalenone varied significantly between men with normal and low sperm count in the control group (Table 4). A significant difference in the level of ochratoxin A was observed between normal and abnormal active motility in the control group. 

### 2.2. Estimating the Effect of the Metabolites of Mycotoxins on Sperm Parameters

To estimate the effect of each of the metabolites on the sperm parameters, non-parametric linear regression was conducted given that the metabolites were not normally distributed. The results of both the unadjusted and adjusted models are presented in Table 5. The level of zearalenone varied in men in the control group. A unit increase in the level of zearalenone significantly predicted a 0.0115 unit decrease in sperm count in the unadjusted model for men in the control group. However, when age, BMI, and type of occupation were controlled, the relationship remained inverse but no longer statistically significant. 

Active motility varied in the cases. Holding other factors constant, a one-unit increase in zearalenone decreased active sperm motility by 0.0302 in the cases. A significant association was observed for the cases in total motility. A unit increase in the level of zearalenone decreased total motility in the cases by 0.0500 when other factors were adjusted. Also, a unit increase in ochratoxin A decreased total motility in the cases by 0.0579, holding other factors constant. Morphology varied by the level of ochratoxin A for the cases. In the unadjusted model, a one-unit increase in the level of ochratoxin A decreased morphology by 0.0268 in the cases. An increasing level of zearalenone predicted an increase in sperm volume for the cases, holding other factors constant. 

## 3. Discussion

The main objective of this study was to compare the urinary levels of metabolites of mycotoxins in men with clinical features of male infertility with normal fertile controls as assessed by semen characteristics and history of recent pregnancies by partners. We used the WHO standard [28] to assess sperm quality and to allocate the participants into each group. Three mycotoxin metabolites—zearalenone, ochratoxin A, and deoxyvinelol—were measured in urine samples of the cases and control groups. The results showed no significant difference in levels of these metabolites between the cases and control groups. Although the median levels of zearalenone and deoxyvinelol were higher in the cases of male infertility, the differences were not statistically significant. There has been documentation of the harmful effects of metabolites of mycotoxins in impairing male infertility in animal models [21,25]. However, to the best of our knowledge, this is one of possibly a few studies that have investigated this relationship in humans.

The concentration of mycotoxins in urine samples are 10-fold higher than in urine samples from Spain [29], but far less in urinary samples from China [30]. These high frequencies of detection and concentrations of mycotoxins can be linked to their presence in Nigerian food, poor storage conditions for cereals that favour the growth of these mycotoxins, and poor food safety policy implementation [31,32,33].

We further investigated the relationships between urinary levels of metabolites of mycotoxins and different parameters of sperm quality—sperm count, motility, morphology, and volume—in the cases and control groups. The results showed that increasing levels of zearalenone predicted decreasing sperm count in the bivariate analysis but when controlled for possible confounding variables, in the multivariate analysis, the results were no longer statistically significant. However, the relationship remained minimally inverse. It was of interest that none of the other urinary metabolites showed any significant relationship with sperm count in the bivariate and multivariable analysis in both cases and control groups.

In contrast, the results showed that sperm motility was less with higher levels of these metabolites in the cases. Specifically, higher levels of zearalenone and ochratoxin A significantly decreased sperm motility in the cases. Similarly, an increase in the level of ochratoxin A decreased sperm morphology in the adjusted and unadjusted models for the cases, while an increasing level of zearalenone predicted an increase in sperm volume for the cases. The increasing level of sperm volume with increasing levels of zearalenone is of interest and may suggest a tendency towards increased production of semen. However, increasing volume of semen does not necessarily correlate to higher sperm quality as it may compromise sperm density and motility. Thus, we report that increasing urinary levels of metabolites of mycotoxins significantly reduces sperm quality, especially sperm motility, and morphology and may not have significant effects on sperm count in humans. 

To date, there has been a dearth of substantive literature on the effects of mycotoxins on sperm quality in humans. However, available animal studies indicate that mycotoxins decrease sperm count and overall efficiency in mammals [21], may reduce the fertilizing ability of spermatozoa in animal models [25], impair sperm motility and velocity [34], and decrease overall sperm quality in swine [35]. These effects of mycotoxins on sperm quality in animal studies have been attributed to the associated estrogenic and non-estrogenic disruptor effects [36], reduced testosterone production [37], reduction in chromatin structure of spermatozoa [38], and decreases in anti-oxidant assimilation [21]. However, it remains critical and important to test the nature of these relationships in humans. 

This study has both strengths and weaknesses. The major strength of the study is the use of a human model rather than an animal model, being one of a few studies that have ever investigated the effects of mycotoxins on human sperm quality and male infertility. Additionally, the prospective and multi-centre study design enabled the use of a large sample size for both cases and controls, while the use of LCMS for assaying the mycotoxins increased the accuracy of measurement and the internal validity of the study. 

The weakness of the study arises from the fact that some metabolites of mycotoxins such as aflatoxins A and B were not assayed. This reduced the ability of the study to investigate the potential effects of all mycotoxins associated with male infertility and sperm quality. Furthermore, we allocated cases and controls on the basis of fertility reports, i.e., the attainment of recent pregnancies by the men who were listed as controls. However, the semen count and quality of the controls were not always perfect when interpreted against the WHO-recommended standards. Some men listed as controls whose wives were reported as recently pregnant had poor semen parameters. This is not unexpected since it is well known that men whose sperm parameters fall below the WHO standards can still attain pregnancies [39,40,41]. However, our analysis of sperm quality differentially in cases and controls enabled us to account for this challenge. Nevertheless, this study provides a guideline for more extensive studies of the relationship between exposure to mycotoxins and the risk of male infertility in an African population.

## 4. Conclusions

We conclude that exposure to mycotoxins reduces the quality of spermatozoa (motility and morphology) in Nigerian men but may have no effect on sperm count. Efforts to reduce the exposure of men to mycotoxins are required to improve sperm quality and reduce the rate of male infertility in the country.

## 5. Material and Methods

Solid-phase extraction (SPE) cartridges (SUPELCO HLB, 500 mg, 12 mL) and mycotoxin standards (zearalenone, ochratoxin A, and deoxyvinelol) were obtained from Sigma Aldrich. Individual stock solutions of all analytes were prepared to obtain 100 mg/L methanol, and 50 µg/L working solutions containing all mycotoxins were prepared by diluting the individual solutions with methanol. All standards were stored in the dark at −20 °C. 

### 5.1. Study Design and Population

This study was part of a comprehensive study to investigate the plausible role of environmental contaminants associated with male infertility. It was designed as a case-control study that compared urinary levels of mycotoxins between infertile men with poor-quality spermatozoa with men reported as fertile with normal sperm quality.

The cases were men diagnosed with azoospermia (absence of spermatozoa in semen) and oligospermia (low sperm count) as well as those with various categories of poor-quality spermatozoa (including poor sperm motility) in the hospitals. They were men attending the infertility clinics in the five participating hospitals who were requested to carry out seminal fluid analysis as part of infertility investigations. The controls were men with normal sperm count and sperm quality who attended antenatal clinics with their wives or who reported recent pregnancies (within 6 months) with their partners/spouses. None of the men were being treated for infertility at the time of the study and none were on specific medications that could affect semen production and quality. All men with co-morbidities such as diabetes mellitus, hypertension, and urinary tract infections/diseases or who were receiving medications for these diseases were excluded from the study.

### 5.2. Sample Size

The sample size was calculated to recruit 154 infertile men aged 18–59 years (cases), in comparison to 136 fertile men aged 18–59 years (controls). The sample size was determined based on the prevalence of infertility of 23% in the study area [42], to enable the detection of a difference with an odds ratio of two, a statistical power of 90%, and a 5% maximum error of the estimate (*p* < 0.05). The study participants were recruited from two teaching hospitals [University of Medical Sciences Teaching Hospital (UNIMEDTH) and the University of Benin Teaching Hospital (UBTH)]; one secondary care hospital (Central Hospital, Benin City, Nigeria); and two private hospitals that provide tertiary infertility treatment (Abel Guobadia Specialist Hospital and the Graceland Specialist Hospital, both in Benin City). Apart from UNIMEDTH which is located in Ondo about 200 km from Benin City, three of the four hospitals in Benin are located within 2 km of each other. The distribution of the sample by the study sites is shown in Table 6.

### 5.3. Data Collection

Research teams were constituted in each hospital that recruited participants and collected data from participants, while the central coordinating team was located at the Abel Guobadia Hospital. 

A protocol was designed to collect information from cases and controls as they were identified from the infertility clinics of the hospitals. The study participants were recruited when they first visited the infertility clinics. After completion of the routines at the first clinic visit, the male partners were invited into another room where the purpose and details of the study (risks and benefits) were provided to them. They were requested to complete a study protocol as well as provide urine and blood samples. Only those who voluntarily accepted to participate in the fully explained study were finally recruited.

The study protocol included sections on socio-demographic variables, history of reproductive health dysfunctions, possible exposures to harmful products, behavioural patterns (e.g., smoking, alcohol use, etc.), results of semen analysis, and hormone assays. Finally, sections were left in the protocol for the results of mycotoxins estimated from urine samples. Midstream urine was taken from cases and controls in clean sterile bottles. The samples were filtered with a 0.45 membrane filter to remove impurities. Samples were collected from the hospitals between December 2020 and November 2021. The semen samples were analysed immediately at the collating hospitals. By contrast, the filtered urine samples were forwarded to the central coordinating office at the Abel Guobadia Specialist Hospital, stored, and frozen at −40 °C until they were ready for analysis. 

### 5.4. Semen Collection and Analysis

Semen samples were collected by masturbation from both cases and controls after at least three days of sexual abstinence. The samples were analysed according to the recommended WHO standards: (1) macroscopically for liquefaction, viscosity, appearance, volume, and pH; and (2) microscopically for total count, motility, viability, and morphology. We assigned normal versus abnormal sperm analysis according to the 2021 WHO criteria [28] as follows: (1) volume ≥ 1.4 mls; (2) total sperm count ≥ 39 million; (3) motility ≥ 42%; (4) viability ≥ 54%; and (5) morphology ≥ 4%.

### 5.5. Analysis of Metabolites of Mycotoxins from Filtered Urine Samples

#### 5.5.1. Solid-Phase Extraction (SPE) 

Filtered urine samples (5 mL) spiked with 1 mL of 50 µg/L of each mycotoxin were extracted using solid-phase extraction (SPE) technique viz a viz the introduction of 5 mL of the sample into SPE cartridges preconditioned with 1 mL of MeOH and 1 mL of H_2_O. The loaded cartridge was washed with 1 mL water, vacuum dried, and eluted using 1 mL each of 2% formic acid MeOH and H_2_O. Then, the samples were dried under vacuum and reconstituted with 1 mL of 0.1% formic acid MeOH/H_2_O (50:50). The reconstituted samples were filtered using 0.22 syringe filter for LCMS analysis.

#### 5.5.2. Liquid Chromatography–Mass Spectrometry (LCMS) Method

The biochemical analysis of metabolites of mycotoxins from the urine samples was undertaken for 179 respondents (case n = 87 and control n = 92) at the Institute for Nanotechnology and Water Sustainability (iNanoWS), University of South Africa (UNISA), using a quadrupole Liquid Chromatography–Mass Spectrometer (LCMS). The separation of the analytes was carried out using a Dionex Ultimate 3000 UHPLC system (Dionex Softron GmbH, Dornierstr. 4, Germany) equipped with a reversed-phase C18 analytical column of 100 mm × 2.1 mm and 1.7 µm particle size (Acquity UPLC^®^ BEH, Waters, Ireland). Column temperature was maintained at 35 °C. The injected sample volume was 5 µL. Mobile phases A and B were water and methanol with 0.1% formic acid, respectively. The optimized chromatographic method was programmed as follows: initial mobile phase composition (2% B) kept constant for 1 min, followed by a linear gradient from 2% B to 100% B for 9 min, kept 100% B for 2 min, and then dropped back to 2% B 12.1 min and kept constant at 2%B for 2 min. The flow rate used was 0.3 mL/min and the total run time was 14 min. This UHPLC system was connected to an Impact II Bruker ultrahigh-resolution quadrupole time-of-flight mass spectrometer (Bruker Daltonics GmbH Fahrenheitstr. 4, Bremen, Germany) equipped with electrospray ionisation, operating in positive ion mode. LC/MS accurate mass spectra were recorded across the range 50–1600 *m*/*z*. The data recorded were processed with Bruker Compass Data Analysis 4.3 software. Accurate mass measurements of each peak from the extracted ion chromatograms were obtained by means of a sodium formate calibrant solution delivered by a KdScientific external pump. The instrument was operated in full-scan mode, except in those cases where automated MS–MS was necessary to discriminate isobars/isomers, as well as for identification of selected compounds and degradation products as explained in the results.

Using this method, the metabolites of mycotoxins analysed were zearalenone, ochratoxin A, and deoxynivalenol (Figure 2A–C).

### 5.6. Validation

All analytical parameters obtained (recoveries, limits of detection, and quantification and linearity) were in accordance with the limits established by European Commission Decision 2002/657/EC [43] (Table 2). Recoveries obtained ranged from 84 to 122% at 100 µg/L for all the mycotoxin metabolites analysed. This falls within the required standard range of 80–120% or wider. 

Calibration curves reveal good linearity of the different concentration ranges for each of the mycotoxin as shown in Table 7 with correlation coefficients (R^2^) between 0.998 and 0.999. Finally, LOD values ranged from 0.16 to 0.61 µg/L, while LOQ ranged from 0.48 to 1.84 µg/L.

### 5.7. Data Analysis

All the analyses were conducted with Stata 17 for Windows. The distributions of the study population by their socio-demographic and behavioural characteristics were presented using percentages for categorical variables and medians with inter-quartile ranges for continuous variables. To test significant association for categorical variables for the case and control groups, the chi-square test was used, and Fisher’s exact test was used when there were cells with counts less than 5. A *t*-test was used for normally distributed characteristics such as weight, and a non-parametric technique, the Mann–Whitney U test, was used for the other continuous variables that were not normally distributed.

The sperm parameters (count, active motility, total motility, morphology, and volume) and the metabolites of mycotoxins (zearalenone ochratoxin A, and deoxyvinelol) were continuous variables and none was normally distributed; thus, medians with inter-quartile ranges and the Mann–Whitney U test were used to present their distribution and test for significant differences between the case and control groups. To estimate the effect of the metabolites of mycotoxins (zearalenone, ochratoxin A, and deoxyvinelol) on the sperm parameters, non-parametric linear regression was used. Unadjusted and adjusted models were estimated. Due to the small sample size for the metabolites, a few variables that are considered confounders, drawing from past studies, such as age, body mass index, and type of occupation, were controlled in the adjusted regression models. Bootstrap was used to estimate the standard errors. All analyses were conducted at a statistical significance level of 0.05 with a 95% confidence interval. 

## Figures and Tables

**Table 1:** Description of the study population by socio-demographic and behavioural characteristics.

Variable	Case (Infertile) N = 136	Control (Fertile) N = 154	*p*-Value
	Median (inter-quartile range)		
Age (n = 290)	40 (10)	40 (9)	0.4253
Weight (n = 288)	81 (21.5)	80 (22)	0.4177
Height (n = 287)	1.72 (0.22)	1.75 (0.28)	0.8271
Body mass index (BMI) (n = 287)	27 (6.79)	26 (6.65)	0.0216 *
	Frequency (percentage)		
Marital status (n = 277)			
Single	11 (8.33)	13 (8.97)	
Married	121 (91.67)	132 (91.03)	0.852
Religion			
Muslim	14 (7.79)	12 (7.79)	
Christian	118 (86.76)	138 (89.61)	
Traditional/other	4 (2.94)	4 (2.60)	0.760
Education			
Primary	7 (5.15)	7 (4.55)	
Secondary	39 (28.68)	35 (22.73)	
Tertiary	86 (63.24)	107 (69.48)	
Other	4 (2.94)	5 (3.25)	0.676
Occupation			
Agriculture	5 (3.68)	5 (3.25)	
Business	38 (27.94)	47 (30.52)	
skilled manual	15 (11.03)	19 (12.34)	
blue collar	19 (13.97)	22 (14.29)	
Professional	28 (20.59)	24 (15.58)	
civil servant	13 (9.56)	24 (15.58)	
Others	18 (13.24)	13 (8.44)	0.553
Frequency of alcohol intake (n = 281)			
Always	8 (6.06)	4 (2.68)	
Often	13 (9.85)	14 (9.40)	
Occasionally	61 (46.21)	73 (48.99)	
Do not take	50 (37.88)	58 (38.93)	0.585
Frequency of smoking cigarettes (n = 268)			
Often	6 (4.69)	1 (0.71)	
Occasionally	4 (3.13)	7 (5.00)	
Do not take	118 (92.19)	132 (94.29)	0.097

Note: * *p* < 0.05.

**Table 2:** Descriptive analysis of sperm parameters (case, control, and total population).

Fertility Status	Sperm Count	Active Motility	Total Motility	Morphology	Volume
**Case** (median)	7	10	20	20	2
IQR	16.8	24	31	38	1
Min	0	0	0	0	0.5
Max	76	100	309	80	7.5
**Control** (median)	46	40	60	57	3
IQR	26	18	17	18	1.5
Min	4.6	9	27	17	0.7
Max	143	85	95	82	7.5
**Total** (median)	28	30	50	48	2.5
IQR	40	29	38	40	1.2
Min	0	0	0	0	0.5
Max	143	100	309	82	7.5
*p*-value	<0.001	<0.001	<0.001	<0.001	0.0060

Note: *p*-value is from Mann–Whitney test; IQR—inter-quartile range.

**Table 3:** Distribution of the metabolites of mycotoxins by fertility status.

Fertility Status	Zearalenone (N = 179) Median (IQR) [Range]	Ochratoxin A (N = 179) Median (IQR) [Range]	Deoxyvinelol (N = 179) Median (IQR) [Range]
**Cases**	326 (550) [0–989.22]	41 (144) [0–2128]	0 (374.58) [0–1236.95]
**Controls**	298.5 (663) [0–2926.32]	46.5 (87) [0–2381]	0 (358.835) [0–1380.53]
Total	314 (642) [0–2926.32]	46 (131) [0–2381]	0 (368) [0–1380.53]
*p*-value	0.9883	0.5588	0.5155

Note: IQR—inter-quartile range. The *p*-value is from the Mann–Whitney U test.

**Table 4:** Variation in the levels of the metabolites of mycotoxins by sperm parameters (case versus control).

Metabolite	Median (IQR) [Min-Max]		*p*-Value	Median (IQR) [Min-Max]		*p*-Value
	Case			Control		
**Sperm count**	Normal	Low		Normal	Low	
	N = 5	N = 82		N = 54	N = 38	
Zearalenone	334 (326) [0–989.22]	321 (537) [0–984.56]	0.5645	223 (459) [0–975.88]	376 (691) [0–986.57	0.0124 *
Ochratoxin A	81 (130) [0–156]	39.5 (144) [0–2128]	0.9696	45 (87) 0–2381]	49 (87) [0–2352]	0.8120
Deoxyvinelol	0 (418.41) [0–560]	0 (319) [0–1236.95]	0.9125	0 (0) [0–1296.2]	0 (679.89) [0–1380.53]	0.1995
	**Normal**	**Abnormal**		**Normal**	**Abnormal**	
**Active motility**	N = 14	N = 69		N = 76	N = 16	
Zearalenone	253.5 (359) [0–950.7]	364 (713) [0–989.22]	0.3212	289.66 (612.5) [0–986.57]	315.5 (609.5) [0–970.47]	0.2195
Ochratoxin A	39.5 (94) [0–825]	32 (150) [0–2128]	0.6603	28.5 (80.5) [0–2352]	80.5 (643.5) [0–2381]	0.0430 *
Deoxyvinelol	0 (82.61) [0–1236.95]	0 (418.41) [0–1210.26]	0.8511	0 (207.835) [0–1380.53]	0 (631.47) [0–791]	0.8204
**Total motility**	N = 24	N = 62		N = 89	N = 3	
Zearalenone	303 (711.81) [0–989.22]	326.5 (537) [0–984.56]	0.9883	298 (642) [0–986.57]	298 (581) [192–773]	0.7643
Ochratoxin A	0 (101) [0–825]	72.5 (156) [0–2128]	0.1585	43 (87) [0–2381]	77 (2109) [52–2161]	0.1456
Deoxyvinelol	0 (852.265) [0–1236.95]	0 (36) [0–1210.26]	0.1836	0 (368) [0–1380.53]	0 (0) [0–0]	0.2804
**Morphology**	N = 60	N = 23		N = 154	N = 0	
Zearalenone	291 (543.5) [0–989.22]	327 (493) [0–976.53]	0.6361	298 (649.5) [0–986.57]	-	-
Ochratoxin A	30.5 (103) [0–2128]	101 (466.99) [0–1926]	0.1385	46.5 (87) [0–2381]	-	-
Deoxyvinelol	0 (718) [0–1236.95]	0 (0) [0–1074]	0.1265	0 (358.835) [0–1380.53]	-	-
**Volume**	N = 74	N = 10		N = 85	N = 5	
Zearalenone	321 (550) [0–989.22]	415.5 (542) [0–984.56]	0.3612	299 (669) [0–986.57]	160 (332) [0–476]	0.3023
Ochratoxin A	33.5 (144) [0–2128]	95 (763) [0–1331]	0.2080	46 (87) [0–2381]	70 (70) [0–1457]	0.7918
Deoxyvinelol	0 (374.58) [0–1236.95]	0 (0) [0–822]	0.3991	0 (416.75) [0–1380.53]	0 (0) [0–0]	0.1525

Note: * *p* < 0.05.

**Table 5:** Regression analysis estimating the marginal effect of the metabolites on sperm parameters.

Metabolites.	Case		Control	
	Unadjusted (SE) [95% CI]	Adjusted (SE) [95% CI]	Unadjusted (SE) [95% CI]	Adjusted (SE) [95% CI]
**Sperm count**				
Zearalenone	0.0081 (0.0050) [−0.0018–0.0180]	0.0041 (0.0090) [−0.0135–0.0217]	−0.0115 (0.0050) [−0.0213–−0.0016] *	−0.0010 (0.0190) [−0.0382–0.0362]
Ochratoxin A	0.0003 (0.0026) [−0.0049–0.0055]	−0.0085 (0.0177) [−0.0431–0.0331]	0.0045 (0.0086) [−0.0124–0.0261]	0.0323 (0.0589) [−0.0832–0.1479]
Deoxyvinelol	0.0124 (0.0075) [−0.0023–0.0271]	−0.0410 (0.1162) [−0.2688–0.1867]	−0.0107 (0.0057) [−0.0219–0.0004]	0.0360 (0.1224) [−0.2040–0.2759]
**Active motility**				
Zearalenone	−0.0006 (0.0051) [−0.0107–0.0094]	−0.0302 (0.0089) [−0.0475–−0.0130] **	−0.0065 (0.0039) [−0.0140–0.0011]	−0.0012 (0.0126) [−0.0259–0.0236]
Ochratoxin A	−0.0037 (0.0028) [−0.0092–0.0019]	−0.0189 (0.0176) [−0.0533–0.0157]	−0.0042 (0.0040) [−0.0120–0.0037]	−0.0051 (0.0367) [−0.0770–0.0668]
Deoxyvinelol	0.0023 (0.0037) [−0.0051–0.0096]	−0.0161 (0.0733) [−0.1598–0.1275]	0.0021 (0.0091) [−0.0157–0.0199]	0.0194 (0.0418) [−0.0624–0.1013]
**Total motility**				
Zearalenone	0.0097 (0.0189) [−0.0274–0.0468]	−0.0500 (0.0212) [−0.0915–−0.0085] *	−0.0012 (0.0029) [−0.0069–0.0045]	−0.0040 (0.0117) [−0.0269–0.0189]
Ochratoxin A	−0.0106 (0.0060) [−0.0223–0.0010]	−0.0579 (0.0255) [−0.1079–−0.0079] *	−0.0041 (0.0028) [−0.0097–0.0014]	−0.0028 (0.0225) [−0.0470–0.0414]
Deoxyvinelol	0.0254 (0.0183) [−0.0103–0.0612]	−0.0130 (0.1033) [−0.2155–0.1895]	−0.0017 (0.0026) [−0.0068–0.0035]	−0.0018 (0.0677) [−0.1344–0.1308]
**Morphology**				
Zearalenone	−0.0066 (0.0091) [−0.0244–0.0112]	−0.0079 (0.0151) [−0.0375–0.0217]	0.0042 (0.0042) [−0.0040–0.0123]	0.0003 (0.0115) [−0.0223–0.0230]
Ochratoxin A	−0.0268 (0.0106) [−0.0475–−0.0061] *	−0.0417 (0.0284) [−0.0975–0.0140]	−0.0007 (0.0137) [−0.0276–0.0262]	−0.0161 (0.0271) [−0.0693–0.0371]
Deoxyvinelol	−0.0047 (0.0055) [−0.0156–0.0062]	−0.0701 (0.1876) [−0.4378–0.2977]	0.0007 (0.0039) [−0.0069–0.0083]	0.0036 (0.0618) [−0.1175–0.1248]
**Volume**				
Zearalenone	0.0002 (0.0003) [−0.0003–0.0008]	0.0023 (0.0005) [0.0012–0.0033] ***	−0.0001 (0.0003) [−0.0008–0.0006]	−0.0001 (0.0009) [−0.0018–0.0017]
Ochratoxin A	−0.0001 (0.0002) [−0.0005–0.0003]	0.0006 (0.0022) [−0.0037–0.0048]	0.0002 (0.0019) [−0.0035–0.0038]	−0.0004 (0.0020) [−0.0043–0.0035]
Deoxyvinelol	0.0037 (0.0050) [−0.0061–0.0135]	0.0065 (0.0603) [−0.1118–0.1247]	2.54 × 10^−6^ (0.0057) [−0.0112–0.0112]	0.0015 (0.0042) [−0.0068–0.0097]

Note: Effect estimates are averages of derivatives. SE is Bootstrap standard error was used because of the small sample size. * *p* < 0.05; ** *p* < 0.01; *** *p* < 0.001.

**Table 6:** Participating hospitals.

Hospital Code	Freq.	Percent
Abel Guobadia	18	6.2
Central Hospital	33	11.4
Graceland	43	14.8
(UBTH)	130	44.8
(UNIMEDTH)	66	22.8
Total	290	

**Table 7:** Validation parameters.

Mycotoxin	Retention Time (min)	Linear Range *	Linearity R^2^	LOD *	LOQ *	% Recovery	RSD
**DON**	4.9	0.1–500	0.999	0.61	1.84	105.16	3.09
**ZEA**	5.9	1–1000	0.999	0.23	0.71	84.49	3.93
**OTA**	6.8	1–1000	0.998	0.16	0.48	122.44	1.61

RSD = relative standard deviation; * = μg/L; LOD = limit of detection; LOQ = limit of quantification.

## Data Availability

Data are available on request from the corresponding author.
